# Overspecification of color, pattern, and size: salience, absoluteness, and consistency

**DOI:** 10.3389/fpsyg.2015.01703

**Published:** 2015-11-06

**Authors:** Sammie Tarenskeen, Mirjam Broersma, Bart Geurts

**Affiliations:** ^1^Department of Philosophy, Radboud UniversityNijmegen, Netherlands; ^2^Centre for Language Studies, Radboud UniversityNijmegen, Netherlands; ^3^Max Planck Institute for PsycholinguisticsNijmegen, Netherlands

**Keywords:** referential overspecification, attribute selection, color, salience, absoluteness, consistent responses

## Abstract

The rates of overspecification of color, pattern, and size are compared, to investigate how salience and absoluteness contribute to the production of overspecification. Color and pattern are absolute and salient attributes, whereas size is relative and less salient. Additionally, a tendency toward consistent responses is assessed. Using a within-participants design, we find similar rates of color and pattern overspecification, which are both higher than the rate of size overspecification. Using a between-participants design, however, we find similar rates of pattern and size overspecification, which are both lower than the rate of color overspecification. This indicates that although many speakers are more likely to include color than pattern (probably because color is more salient), they may also treat pattern like color due to a tendency toward consistency. We find no increase in size overspecification when the salience of size is increased, suggesting that speakers are more likely to include absolute than relative attributes. However, we do find an increase in size overspecification when mentioning the attributes is triggered, which again shows that speakers tend to refer in a consistent manner, and that there are circumstances in which even size overspecification is frequently produced.

## 1. Introduction

When speakers refer to objects, they do not always limit themselves to giving information that is strictly necessary for the addressee to identify the referent. In other words, they sometimes produce *overspecification* instead of *minimal specification* (e.g., Pechmann, 1989; Engelhardt et al., 2006; Arts et al., 2011b). Imagine, for example, a speaker requesting her addressee to pass her a yellow cup, which happens to be surrounded by blue plates and bowls. Although the speaker need not include a color adjective to enable her addressee to identify the referent, because there is only one cup present, experimental work suggests that she would be more likely to utter (1-b) than (1-a) in this situation, and hence, to produce *color* overspecification.

(1) a. Please pass me the cup.
b. Please pass me the *yellow* cup.

Experimental findings suggest that there is something special about color in reference: including color is preferred over including various other attributes, most notably size. When it is necessary to include either color or size to get a unique description of the referent, color is more often included than size (Belke and Meyer, 2002). Color is also more likely to be included redundantly than size: for example, when referring to a *small* yellow cup surrounded by *big* cups in yellow, red, and green, many speakers will not only select size, which is both necessary and sufficient for identification of the referent, but also color, which is neither necessary nor sufficient (Pechmann, 1989). When referring to an object that is unique in its type, as in the situation above, speakers often include color as well (Koolen et al., 2013), even though no modification (e.g., an adjective) is needed at all in that case. Most extremely, even when all objects in the visual context have the same color as the referent, color is sometimes mentioned (Mangold and Pobel, 1988; Belke and Meyer, 2002; Koolen et al., 2015).

In this paper, we investigate the seemingly special status of color in reference production, and in overspecification in particular. We do this by comparing color with two other attributes: pattern and size. Whereas color and size overspecification have been investigated before, the study of reference to pattern is virtually unexplored. Pattern is an interesting attribute because it is like color—but unlike size—in being both *salient* and *absolute*. As these two factors have been suggested to explain why speakers produce color overspecification, comparing the three attributes will enable us to systematically tease apart, for the first time, the effect of the two factors on the tendencies to include different attributes redundantly.

We present a series of four language production experiments. In our first experiment, we compare the rates of color overspecification with the corresponding rates of pattern and size overspecification. In one follow-up experiment, we then assess the effect of salience and absoluteness. In two other follow-up experiments, we assess the effect of *consistency*, that is, the tendency to reuse previous expressions and constructions, by varying color, pattern, and size both within and between participants, and by triggering selection of the three attributes.

## 2. Salience, absoluteness, and consistency

In this section, we discuss the literature on referential overspecification. In Section 2.1, we introduce the notion of salience as an important factor in attribute selection. The role of salience and absoluteness in the preference that speakers appear to have for including color is elaborated on in Section 2.2. In Section 2.3, we discuss experimental work on the speakers' tendency to behave consistently. Finally, we introduce the series of experiments that we conducted in more detail in Section 2.4.

### 2.1. Salience and overspecification

A question in the research of referring expressions production that has received much attention lately is how speakers select attributes when producing definite descriptions (for a recent overview, see van Deemter et al., 2012). A factor that is currently thought to be central to attribute selection is *salience* (e.g., Gatt, 2007; Arts et al., 2011a; Koolen et al., 2011). An object's attribute can be salient for various reasons, and is then more likely to be selected by a speaker who intends to refer to this object. This may result in overspecification, as salient attributes are not always necessary to enable the addressee to identify the referent.

The basic idea of selecting salient attributes is intuitive: speakers tend to select the attributes according to the degree to which their attention is attracted by them. In the literature on salience and visual perception, visual or perceptual salience is considered to be a property of *objects*, which may be defined in terms of surprise (Itti and Baldi, 2009). Surprise can occur on a low level, for example, when an object is unique on one or more dimensions (Treisman and Gelade, 1980), such as a blue round candy among red cubic candies. It can also occur on a higher level, induced by world knowledge (Franke, 2012): a blue banana will in general be more salient than a yellow banana.

In the literature on reference production, it is assumed (often implicitly) that not only objects, but also *attributes* of objects vary in salience (e.g., Davies and Katsos, 2013). Attributes that are unique in a given context, like color and shape in the candies example above, may be salient, and attributes that are surprising due to world knowledge, such as the color of a blue banana, may be salient as well, analogously to factors that determine the salience of objects. Indeed, speakers tend not to include redundant color adjectives when referring to objects strongly associated with a specific color, for instance, the color of a yellow banana (Sedivy, 2003), which is entirely as expected and therefore not particularly salient. If a referent has an unexpected color, however, color overspecification is much more likely to occur (Westerbeek et al., 2014). Davies and Katsos (2013) show that speakers are more likely to produce overspecification when objects have salient attributes than when they do not.

It seems a good idea to select attributes that are salient, not only because it is easy for the speaker, as has often been suggested (Mangold and Pobel, 1988; Davies and Katsos, 2013; Koolen et al., 2013), but also, and perhaps more importantly, from a communicative point of view (cf. Arts et al., 2011b; Koolen et al., 2011; Davies and Katsos, 2013). If an attribute attracts the speaker's attention, it is likely that it will attract the attention of her addressee as well, which probably increases the likelihood that it is useful in the process of identifying the referent. Not all salient attributes are necessary for referent identification, however, and selecting them may therefore result in overspecification. Although the word “overspecification” may have a negative flavor, suggesting that the expression is *too* specific, overspecification need not be cumbersome and may even be beneficial, as the benefits of a strictly redundant but salient attribute in the comprehension process may often outweigh the risk that the addressee is hindered by its redundancy. Indeed, there is evidence that overspecification can speed up the process of referent identification (Sonnenschein and Whitehurst, 1982; Mangold and Pobel, 1988; Paraboni et al., 2007; Arts et al., 2011b; but see Engelhardt et al., 2006, 2011). An eyetracking study on the processing of size and color adjectives suggests that redundant size adjectives may be confusing for addressees, whereas redundant color adjectives are not (Sedivy et al., 1999). Another study on the comprehension of overspecified expressions suggests, moreover, that non-salient redundant attributes are more likely to hinder the addressee than salient redundant attributes (Davies and Katsos, 2013).

In sum, there seems to be a tendency to select salient attributes, even if this results in overspecification. Redundancy can hinder the comprehension process, but as salient attributes are likely to be helpful in referent identification, including a redundant but salient attribute may often be beneficial.

### 2.2. The color preference

The literature suggests that speakers tend to include color more often than other attributes, and that color overspecification is more common than overspecification of other attributes. Two features of color have been argued to contribute to this preference: salience and absoluteness. We will discuss both features in this section. An overview of salience and absoluteness of color, pattern, and size is presented in Table 1.

**Table 1:** **Salience and absoluteness of the three attributes**.

	**Salience**	**Absolute**
Color	High	Yes
Pattern	High	Yes
Size	Experiments 1 and 2: Low	No
	Experiments 3 and 4: High	

#### 2.2.1. Salience

In line with the view that speakers tend to select salient attributes, it has been argued that color is preferred because it is *intrinsically* salient (Arts et al., 2011a; Gatt et al., 2013; Koolen et al., 2013). The common view is that intrinsically salient attributes are noticed immediately, and before other attributes: they are “perceived earlier” (Gatt, 2007) and “immediately grab [the speakers'] attention” (Koolen et al., 2013). It has also been suggested that color is more likely to “pop out” than other attributes (Westerbeek et al., 2014): intuitively, one green candy in a jar surrounded by red ones is more likely to be noticed than one small candy surrounded by big ones, or one cubic candy surrounded by round ones.

Indeed, color is one of the features computed in the earliest stages of human visual processing (Livingstone and Hubel, 1988), and can be considered a primary cue in visual perception. It has been found that objects in a color that is contextually unique can grab the attention in visual search, even if color is irrelevant to the task (Theeuwes, 1992; Turatto and Galfano, 2001). Color also tends to be more helpful in visual search than other attributes, such as size and shape (Williams, 1966; Christ, 1975). Color contrast between items thus seems to be an extremely powerful cue in visual perception. In this respect, color may be different from other visual attributes, and also from non-visual attributes, like material, some of which have been found to be included redundantly less often than color (see Mangold and Pobel, 1988, for shape, Arts et al., 2011b, for size, and Sedivy, 2005, for size and material).

When examining experimental stimuli from previous experiments, however, we observed that colors in experimental stimuli tend to be bright and/or highly contrastive, while differences in size are usually rather modest (e.g., Arts et al., 2011b; Koolen et al., 2011). We argue, then, that previous findings do not necessarily show that color is preferred over size due to a difference in salience. Rather, the specific colors and color contrasts used in those experiments may have been more salient than the size contrasts used, resulting in higher rates of color overspecification. Recently, the preference for color over size was found to disappear when the size contrast between the referent and other objects was increased (van Gompel et al., 2014). Along the same lines, speakers may be less inclined to produce color overspecification when the color contrast is low or when colors are not particularly vivid than when colors are bright and contrastive (Tarenskeen et al., in preparation). In sum, it is not evident that, for example, a pale blue candy surrounded by mint green ones is more likely to get the attention than a huge candy surrounded by tiny ones.

In the study conducted by van Gompel et al. (2014), *competition* between color and size was investigated. In the condition relevant for our study, the referent was different from the other objects in the array in color and size but not in type. For example, the referent was a small red candle and the other objects were a big blue and a big black candle. When the size contrast was low, participants included color but not size in 79% of the cases, and size but not color in only 2% of the cases. When the contrast was high, however, color but not size was included in only 27% of the cases, while the rate of referring expressions including size but not color increased to 23%. Importantly, it was always necessary to include either color or size. Hence, overspecification occurred only when *both* color and size were included. This set-up is suitable for studying attribute preferences, but not for comparing attributes with respect to how likely they are to be added redundantly, which is the aim of the present study. To be able to compare the rates of color, pattern, and size overspecification, we present participants with arrays in which the referent is unique in its type (for example, if the referent is a dress, none of the other objects in the array is a dress). Thus, adding an extra attribute always results in overspecification. As van Gompel et al. (2014), we manipulate the size contrast between the referent and surrounding objects. While they investigate the effect of size contrast on the choice for including size vs. color, we assess the effect of size contrast on the production of size overspecification.

While we vary the salience of size, we keep the two other attributes constant in being high in salience. Unlike color and size, pattern is virtually unexplored in the literature on reference production. In the only study investigating pattern in reference production, Gatt et al. (2013) found that speakers prefer color over both pattern and size. As in van Gompel et al.'s study, however, they investigated competition between attributes, using arrays in which the referent was not unique in its type. Moreover, they used a single superimposed shape (a circle, a diamond, or a square) on a brightly colored picture as patterns, e.g., a green bottle with a circle-shaped patch on it. Such patterns are probably not very salient, and pictures with one little figure would not normally be called “patterned”. The use of striking colors may have decreased the salience of pattern even more. This thus leaves the crucial question open whether speakers are also more likely to produce color than pattern overspecification in a situation where pictures have salient patterns but no other salient attributes. The present study aims to address this question by depicting patterned objects which are completely striped or spotted and do not have any other striking attributes. If color overspecification is produced frequently because of its intrinsic salience, a high rate of pattern overspecification is expected too, as pattern may be highly salient as well. On the other hand, a high rate of size overspecification is only expected if size is made salient. In Section 2.4, we elaborate on this further.

#### 2.2.2. Absoluteness

According to Pechmann (1989) and Belke and Meyer (2002), speakers tend to select color before size because color is an absolute attribute, whereas size is relative^1^. That is, a speaker need not take into account objects surrounding the referent in order to determine its color^2^, while she normally has to do this to determine whether the referent is big or small. Pechmann points out that as speech is produced incrementally, the speaker can start to articulate the referent's color while examining the context in order to find out which additional attributes are required for a unique description, which may result in color overspecification. Pechmann's argument is in line with eyetracking results which indicate that speakers often start producing color adjectives before fixating on an item of the same type but a different color in the array (e.g., a blue cup when the referent is a yellow cup), while they rarely start producing size adjectives before fixating on a size-contrastive item (Brown-Schmidt and Konopka, 2011).

Two findings indicate that absoluteness alone does not explain the color preference. First, not all absolute attributes tend to be redundantly included in referring expressions. Although shape is an absolute attribute, shape overspecification has been found to occur less frequently than color overspecification (Mangold and Pobel, 1988; Arts et al., 2011b). In another study, material, which is also an absolute attribute, was included redundantly as infrequently as size, even though size is a relative attribute (Sedivy, 2005).

The second indication that absoluteness alone does not explain the color preference is that size adjectives usually precede both redundant and non-redundant color modifiers (e.g., “the big red car,” Sproat and Shih, 1991; Cinque, 1994), while according to Pechmann's account, redundant color modifiers should in general precede size modifiers (“the red big car”). After all, color overspecification is due to speakers starting their referring expression after selecting color but before selecting size. In Pechmann's production study, speakers of Dutch indeed produced color before size adjectives sometimes, even though they would normally prefer the reverse order (Sproat and Shih, 1991, p. 580). However, in two studies with speakers of German and English, who have the same adjective order preference as speakers of Dutch (Cinque, 1994), color overspecification was produced frequently, but color hardly ever preceded size (Belke, 2006). This indicates that color overspecification is often not due to articulating color adjectives before selecting size, as Pechmann proposes. It is possible, however, that color is normally *selected* before size, without necessarily being *articulated* before selecting size (see also Belke and Meyer, 2002).

Although the distinction between absolute and relative attributes thus cannot entirely explain the asymmetry between color and size, the fact that color is absolute while size is relative is likely to play a role in the preference for color over size in reference. In the present study, we take into account the role of absoluteness by comparing color both to size, which is relative, and to pattern, which is absolute.

### 2.3. Consistency

Our main interest in this paper is in the overspecification of three different attributes that vary in salience and in being absolute or relative: color, pattern, and size. Additionally, we investigate the way in which the rates of overspecification of the three attributes may affect one another. Experimental studies show that speakers have a preference for sticking to previously used expressions and constructions (e.g., Brennan and Clark, 1996; Pickering and Garrod, 2004; Goudbeek and Krahmer, 2012). In this paper, we investigate the relation between this preference and tendencies to include one attribute but not another one. For example, if speakers have a preference for including color but not including size, a preference for consistency may result in a decrease in the rate of color overspecification, or an increase in the rate of size overspecification.

Recently, the attention of some researchers has been attracted by the high amount of variation *across* speakers when producing referring expressions in experimental settings. It was found that machine learning models predict human-produced referring expressions better when they take into account both speaker identity and characteristics of the visual context than when they only use visual characteristics (Viethen and Dale, 2010; see also Mitchell et al., 2011; Ferreira and Paraboni, 2014). Since machine learning models that used speaker identity based their predictions on previously produced referring expressions, this finding suggests not only that speakers strongly differ in their referring behavior, but also that individual speakers tend to be consistent in the way they refer. Indeed, a basic assumption in psychological research is that variation between participants is higher than variation within participants, which is why participants are often modeled as random variables in statistic analyses (e.g., Baayen et al., 2008).

The finding that speakers tend to refer in a consistent way is reminiscent of the well-established tendency to reuse referring expressions that have been used earlier in the conversation by one of the interlocutors. For example, Brennan and Clark (1996) showed that speakers who use a specific term instead of a basic-level term in order to avoid ambiguity, such as “the loafer” in a context with several kinds of shoes, tend to stick to this term even in contexts where the basic-level term would not lead to ambiguity any longer, such as ‘the loafer’ in a context where the loafer is the only shoe. Analogously, speakers were found to reuse constructions for the same referents by including modifiers that were redundant in the current context but necessary in preceding contexts (Van Der Wege, 2009).

More generally, speakers can be primed to include attributes that would normally be dispreferred, such as the orientation of the referent where its color would have been sufficient, too (Goudbeek and Krahmer, 2012). Another study suggests that attribute selection is affected by the linguistic context more than by some visual factors that are often expected to be influential, such as the degree to which the referent's attributes are unique in the visual context, called discriminatory power^3^ (Viethen et al., 2014). They found that learning models of reference production that take into account features of previously produced referring expressions predicted human-produced expressions better than models selecting attributes based on discriminatory power, which is also in line with Gatt et al. (2013). The tendency to reuse words in experimental settings has been found outside the realm of reference as well (see e.g., Alferink and Gullberg, 2014).

In our study, we investigate whether due to a tendency toward consistency, the tendencies to include one attribute but not another can affect one another. We also assess whether, in line with Goudbeek and Krahmer (2012), mentioning the three attributes can trigger even size overspecification, which is normally produced infrequently. Our study is not intended, however, to assess the mechanisms that underpin consistency in reference production. Currently, a debate is going on about those mechanisms. One position is that in dialogue, interlocutors establish *conceptual pacts* (Brennan and Clark, 1996): they reuse referring expressions when talking to the same partner and expect their partner to do the same. This view presupposes that interlocutors keep track of their common ground, that is, the information that is mutually shared between them. According to the alternative account, interlocutors automatically *align* their representations on all linguistic levels (Pickering and Garrod, 2004). The central claim is that interlocutors do not need to keep track of their common ground, memory processes like priming normally being sufficient for proper alignment. That is, interlocutors reuse referring expressions because those expressions are salient due to their being primed by their previous usages. It is uncontroversial that priming is a mechanism present in both language production and comprehension: there is substantial evidence for semantic priming (e.g., Meyer and Schvaneveldt, 1971; Neely, 1976), phonological priming (e.g., Bock, 1986a; Grainger and Ferrand, 1996), and syntactic priming (e.g., Bock, 1986b; Potter and Lombardi, 1998). What researchers in the present debate essentially disagree about, however, is whether interlocutors routinely take into account their common ground when producing and comprehending utterances in a way that goes beyond automatic priming mechanisms (see amongst many others, Brown and Dell, 1978; Lockridge and Brennan, 2002; Pickering and Garrod, 2004; Yoon and Brown-Schmidt, 2014).

In sum, speakers often reuse words and constructions that were used earlier in the discourse, having a preference for consistency. They tend to do this even if there is in fact a good reason to switch to a different construction, like the changed context in Brennan and Clark's (1996) experiment, or the general preference for other attributes than orientation, as in Goudbeek and Krahmer's (2012) experiment. Consistency in reference production may be due to considerations of the interlocutors' common ground or to simple priming mechanisms. However, we are neutral as to what mechanisms may result in the effects we find, although we will discuss some possibilities in Section 7.

### 2.4. The present study

The present study investigates, in the first place, tendencies to include various attributes in referring expressions, even if this results in overspecification, and the way in which salience and absoluteness contribute to these tendencies. In order to do this, we conduct four language production experiments in which speakers use referring expressions to refer to pictures of objects that vary in color, pattern, and size. We compare the proportions of overspecification of the three attributes. Our study is the first to compare attributes such that salience and absoluteness are systematically teased apart. We do this by varying the salience of size between experiments. Throughout the experimental series, we also explore the tendency toward consistent behavior, examining to what extent speakers alternate between including and not including an attribute, and investigating the effect of including necessary attributes on the production of size overspecification in particular.

Experiment 1 is a baseline study in which we investigate the rates of color, pattern, and size overspecification. As discussed in the previous section, color, which has been argued to be “special” with respect to overspecification, is similar to pattern in being salient and absolute (see Table 1). Size, on the other hand, differs from color and pattern in being relative instead of absolute. Further, in Experiment 1, the contrast between big and small items is low and size is hence low in salience. As such, size is different from both color and pattern, in being relative and less salient. If speakers tend to include color because it is salient and absolute, they are expected to include other attributes that are salient and absolute as well. We therefore hypothesize that in comparison to size overspecification, speakers will not only produce more color overspecification, which would be in line with what has been found before (Pechmann, 1989; Belke and Meyer, 2002; Gatt et al., 2013), but also more pattern overspecification.

In Experiment 2, we explore the possibility that in Experiment 1, where a within-participants design is used, the expected tendency toward consistency may lead to an effect of the tendency to include or not include one attribute on the rate of overspecification of another attribute. For example, pattern might be treated like color because the two attributes share characteristics with each other but not with size. Another possibility is that not including size in their utterances will lead some speakers to stop producing color and pattern overspecification as well. In Experiment 2, we investigate the occurrence of such effects in Experiment 1, by varying the three attributes between instead of within participants. If the rates of overspecification tend to affect one another, the pattern of results is expected to change compared to the pattern found in Experiment 1.

In Experiment 3, we delve into the question of how salience and absoluteness contribute to the tendency to include attributes, teasing these two features apart. We make size more salient by increasing the contrast between big and small items. We hypothesize that the rate of size overspecification increases correspondingly, which would indicate that salience is a factor in selecting attributes and producing overspecification. Furthermore, we expect absoluteness to have an effect, too, leading to higher rates of overspecification of the two absolute attributes (color and pattern) than the relative attribute (size).

Experiment 4, finally, investigates whether overspecification of the three attributes is triggered by including non-critical trials which, unlike the critical trials, require color, pattern, or size to be included. The experiment is thus conducted to assess whether the production of overspecification of color, pattern, and even size, can increase due to a tendency toward consistency.

## 3. Experiment 1

In Experiment 1, we vary color, pattern, and size in a within-participants design and compare the rates of overspecification for the three attributes. As color and pattern are salient and absolute while size is less salient and relative, we hypothesize that the rates of color and pattern overspecification will be higher than the rate of size overspecification. We also explore the tendency toward consistency by examining the individual proportions of alternations between overspecification and minimal specification in each condition.

### 3.1. Method

#### 3.1.1. Participants

We tested 18 native speakers of Dutch (14 females, 4 males, mean age 23 years, range 18–27 years) at Radboud University, Nijmegen, the Netherlands. All were volunteers and they received a small fee for their participation. All of them reported not to be colorblind.

#### 3.1.2. Materials

**Figure 1:**
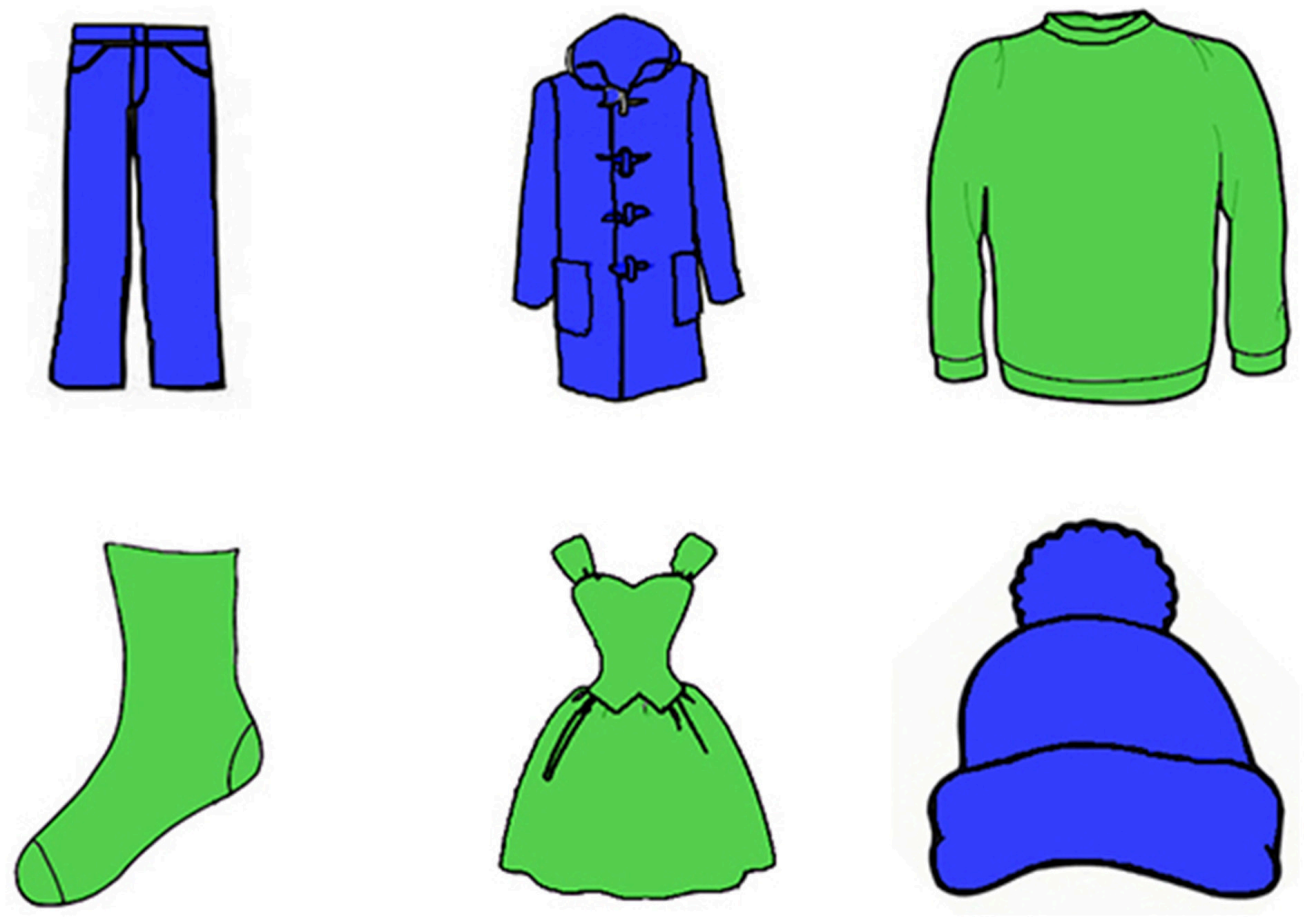
**An array in the Color condition in Experiment 1**.

**Figure 2:**
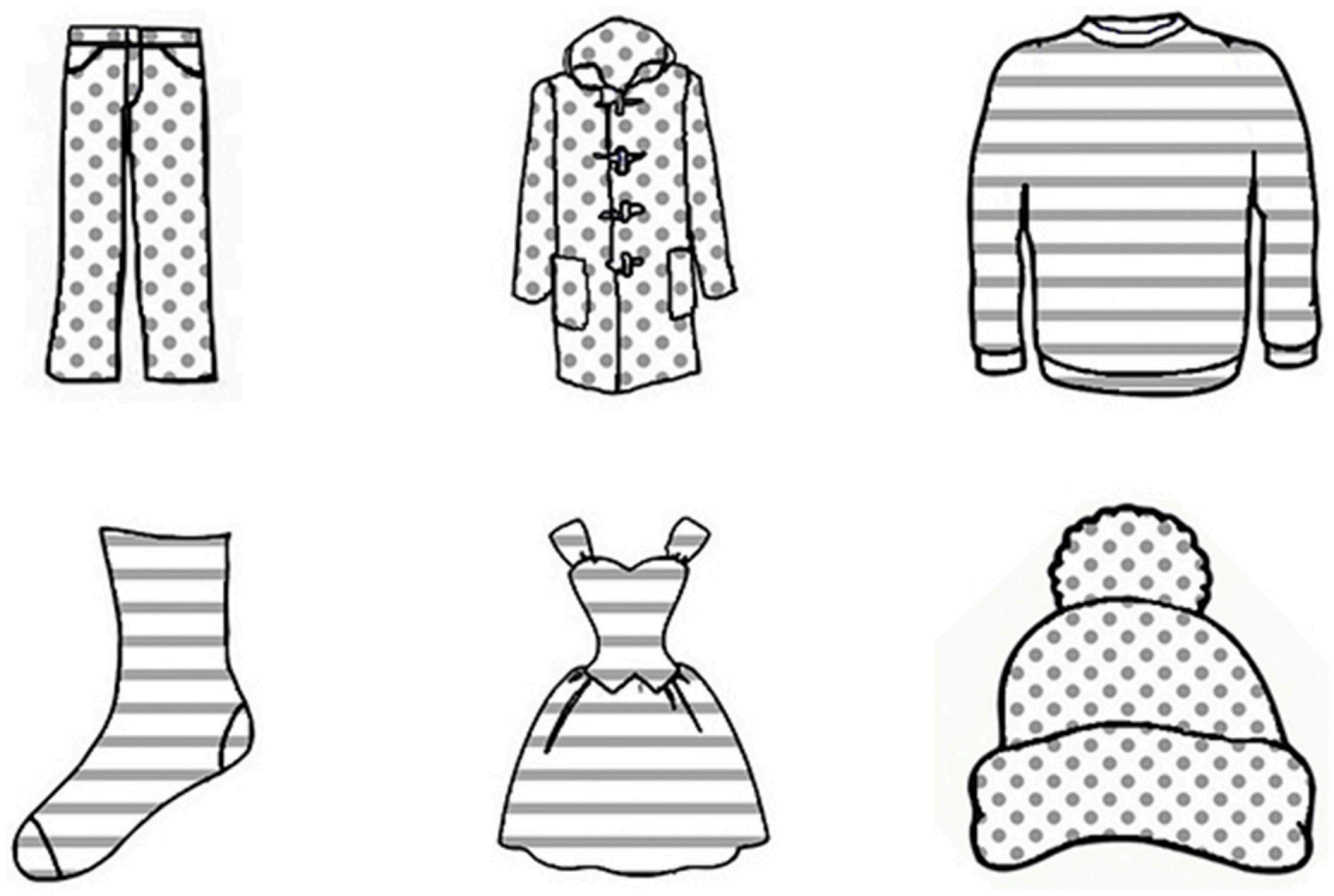
**An array in the Pattern condition in Experiment 1**.

**Figure 3:**
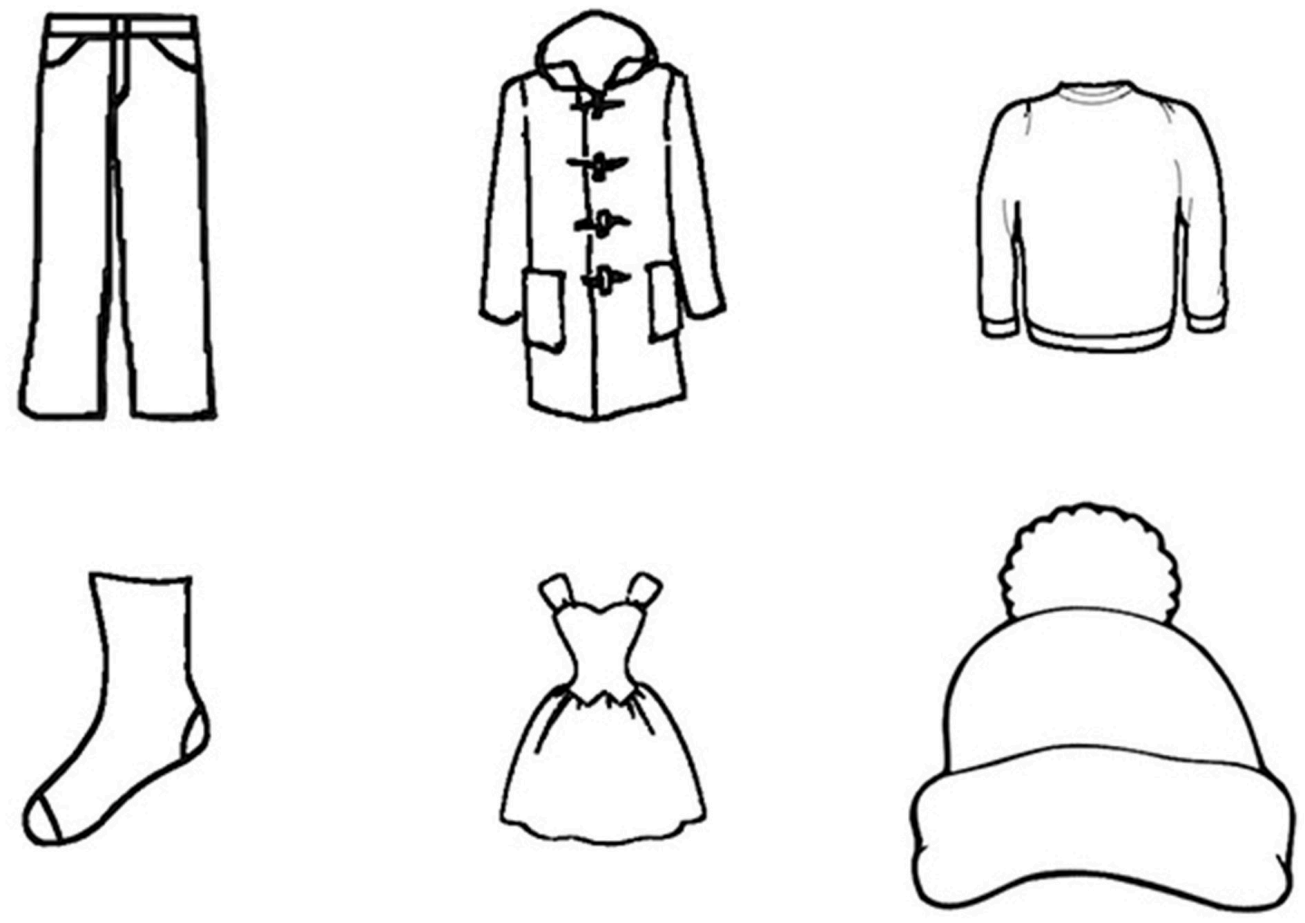
**An array in the Size condition in Experiment 1**.

We used six line drawings of clothes as stimulus materials, which were collected on Google Image. All garments would normally be named by a one-syllabic noun in Dutch. The six pictures were manipulated in order to create variation on the three attributes. Relative size is expressed in Dutch by equivalents of “big” and “small,” which makes it basically a binary attribute. We therefore selected two values of each of the two other attributes, too. The pattern values were striped and spotted, the color values were blue and green, and the size values were big and small, as shown in Figures 1–3. We thus created six variants of each picture. The patterns were clear gray stripes or spots against a white background and the colors were bright, saturated colors. The ratio between the heights of the big and small pictures was 3:2. The experiment was programmed with Presentation software.

We also had filler pictures, which were taken from the Tarrlab Stimulus Repository^4^. There were three types of filler pictures: common objects, like bikes and envelopes (Rossion and Pourtois, 2004), Greebles (Gauthier and Tarr, 1997), and human faces. Greebles are complex and visually similar, which makes them difficult to describe uniquely. So as not to stimulate participants to pay special attention to color, filler pictures were presented in desaturated, inconspicuous colors (common objects) or in gray tones (Greebles).

#### 3.1.3. Design

In critical trials, an array was presented with pictures of six different garments. They were arranged in a 2 (row) × 3 (column) grid. We had three conditions: Color, Pattern, and Size. The objects within an array always varied on exactly one attribute: color, pattern, or size, respectively. In each array, half of the objects had one value (e.g., striped) and the other half had the other value (e.g., spotted). The target object thus shared its value with two other objects. Including a color, pattern or size modifier always resulted in overspecification. Examples of arrays are shown in Figures 1–3.

Attribute was manipulated within participants: each participant received trials from all three conditions. Each of the six objects once acted as target in each of the six possible values, yielding 36 critical trials. All participants saw all critical trials. They also saw 36 trials of each of the three filler types, yielding a total of 144 trials. Eight additional trials were included for practice.

Fillers were included for two reasons: first, to prevent participants from sticking to one syntactic and semantic structure throughout the whole experiment, and second, to hide the purpose of the experiment. There were three types of filler trials. Fillers of the first type consisted of arrays with four pictures of common objects, which were included to elicit unmodified referring expressions, that is, expressions without any adjectives or prepositional phrases. We did not expect modification to occur because basic-level terms were always sufficient and pictures did not have striking or unexpected features. Fillers of the second type were arrays with four pictures of Greebles, which were included to make participants aware that simply naming objects was not always sufficient. Fillers of the third type were arrays with two human faces, which were either of the same gender or of different genders. They were included to elicit variation in the presence of modifiers within a category: modification was necessary when the two people were of the same gender, but unnecessary when they differed in gender.

The order of the trials was pseudorandomised, with the restriction that a trial was always followed by at least two trials in which the target was of a different type of garment. For example, when the target was a sock, the target in the next two trials was never a sock. We did this in order to prevent participants from producing an adjective for the sake of contrast between the referent and the previous referent, which speakers have been shown to do in reference production experiments (see Levelt, 1989, p. 132; Pechmann, 1989, for discussion of this type of factors in reference production). Each participant saw the trials in a unique order.

#### 3.1.4. Procedure

Participants were tested individually in a quiet booth. Their task was to instruct an imaginary addressee to click on one of the pictures, by completing the Dutch equivalent of the sentence “Click on ….” A cross preceding the array indicated the position of the target on the screen. Participants were asked to formulate their instruction in such a way that an addressee would be able to click on the right picture, even if the pictures would be arranged differently on the screen for the addressee than for the participant. This particular instruction was given to prevent them from referring to the location of the pictures on the screen. It took participants about 20 min to complete the task.

### 3.2. Results

Each participant performed 36 critical trials. In two trials, no response was given. The critical trials thus elicited 646 responses. Seventeen responses (2.6%) were removed, because the referent was not the target item, or because the speaker corrected themselves during the articulation of the utterance. The remaining 629 expressions were annotated as overspecified when a color modifier was included in the Color condition, when a pattern modifier was included in the Pattern condition, and when a size modifier was included in the Size condition^5^.

**Figure 4:**
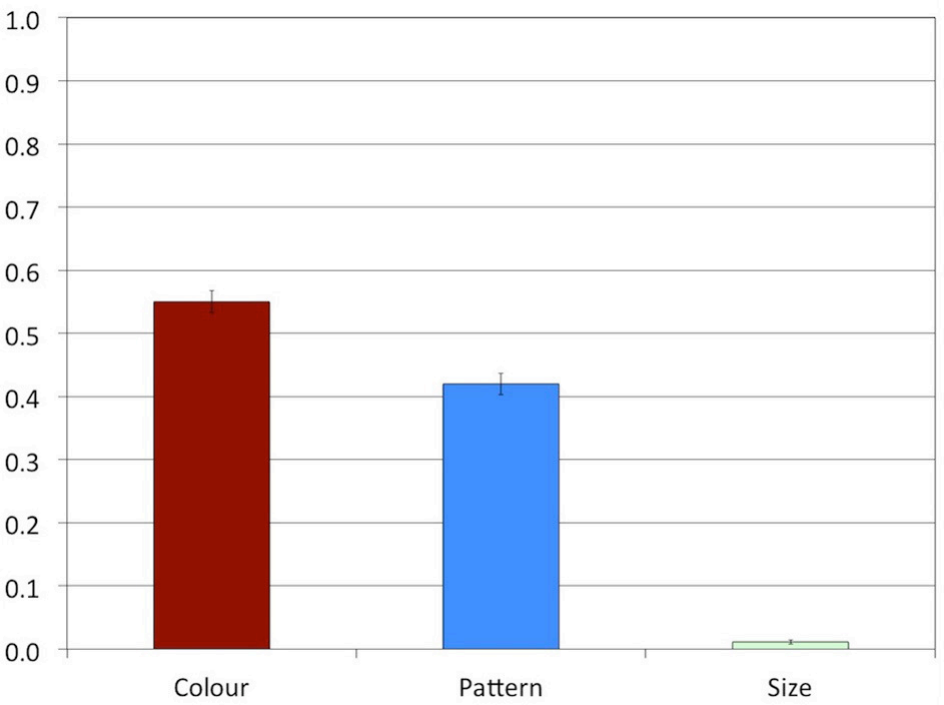
**Experiment 1: Proportions of overspecified referring expressions**. The error bars represent standard errors.

Experiment 1 was conducted to answer the question how likely speakers are to produce overspecification of color, pattern, and size, respectively. We expected that overspecification would be produced more often in the Color and the Pattern conditions than in the Size condition. Indeed, Figure 4 shows that overspecification was produced often in the Color condition (proportion of overspecification: *M* = 0.55, *SD* = 0.50) and in the Pattern condition (*M* = 0.42, *SD* = 0.49), but almost never in the Size condition (*M* = 0.01, *SD* = 0.10).

In this experiment and all the following, Shapiro-Wilk tests indicated that the data were not normally distibuted (*p* < 0.001 in all conditions in all experiments). Hence, we ranked the data and used non-parametric statistics for the analyses. We report mean ranks, denoted by *MR*.

A Friedman's ANOVA indicated a highly significant main effect of Attribute on overspecification, χ^2^(2) = 24.24, *p* < 0.001. In line with our hypothesis, stepwise stepdown comparisons indicated a significant difference between the Pattern (*MR* = 2.17) and Size (*MR* = 1.19) conditions, *p* = 0.005, while the difference between the Pattern and the Color (*MR* = 2.64) conditions was not significant, *p* > 0.1.

**Figure 5:**
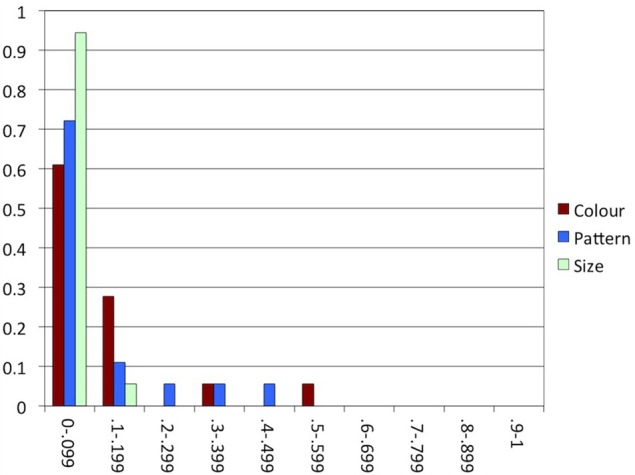
**Experiment 1: The proportion of participants (y-axis) in each range of proportions of alternations in each condition (x-axis)**.

To explore the tendency toward consistent behavior, we counted the number of times that participants included an attribute in a trial but did not include it in the next trial of the same condition, or vice versa. For each participant, we divided this number by the number of trials of the condition −1 (the number of opportunities to alternate). Figure 5 shows the degree of consistency in each condition, indicating that participants tended to behave highly consistently, the majority alternating in less than 10% of the trials within each condition.

### 3.3. Discussion

Experiment 1 indicates that, in line with our expectations, speakers produced substantial rates of color and pattern overspecification, but hardly any size overspecification. Although the rate of color overspecification was numerically higher than the rate of pattern overspecification, this difference was not significant. It seems, then, that color and pattern overspecification are both likely to occur, both attributes being salient and absolute. In line with the literature, we found that speakers were highly consistent within conditions, most of them either producing or not producing overspecification in the majority of the trials.

As was pointed out before, the tendencies to include or not include one attribute may have affected the rate of overspecification of another attribute, due to a tendency toward consistency. It is possible, for example, that a tendency to include color may have triggered the production pattern overspecification, since the two attributes share characteristics with each other but not with size. Another possibility is that the tendency not to include size has resulted in a decrease in overspecification overall.

In Experiment 2, we vary the three attributes between participants, thereby excluding the possibility that the rate of overspecification in one condition affects the rate in another. A change in the pattern of results would therefore indicate that such between-attributes effects took place in Experiment 1, probably due to the tendency toward consistency. A stable pattern, in contrast, would show that the rates of overspecification of the three attributes did not affect one another.

## 4. Experiment 2

In Experiment 2, we vary color, pattern, and size in a between-participants design, in order to find out whether the rates of overspecification in Experiment 1 affected one another, due to a tendency toward consistent behavior. A change in the pattern of results would indicate that such effects occurred, whereas a similar pattern would show that they were absent. Again, we expect a high degree of consistency within speakers.

### 4.1. Method

#### 4.1.1. Participants

We tested 54 participants (43 females, 11 males, mean age 22 years, range 18–31 years) similar to those in Experiment 1^6^. None had participated in the previous experiment.

#### 4.1.2. Materials, design, and procedure

Materials were the same as in Experiment 1. Attribute was now manipulated between participants. Participants were randomly assigned to either of the three conditions: Color, Pattern, or Size, with 18 participants per group. In each condition, there were twelve different critical pictures in each condition (6 pictures ^*^ 2 values of the attribute in that condition). Each picture was presented twice in each experimental session, yielding 24 critical trials in each condition. Participants also received 24 trials of each of the three filler types, yielding a total of 96 trials. Four additional trials were included for practice. Otherwise design and procedure were the same as in Experiment 1.

### 4.2. Results

All participants performed 24 critical trials. Once, no response was given. The critical trials thus elicited 1295 responses. We excluded 28 responses (2.2%) from the analysis as in Experiment 1. The remaining 1267 expressions were annotated as in Experiment 1^7^.

**Figure 6:**
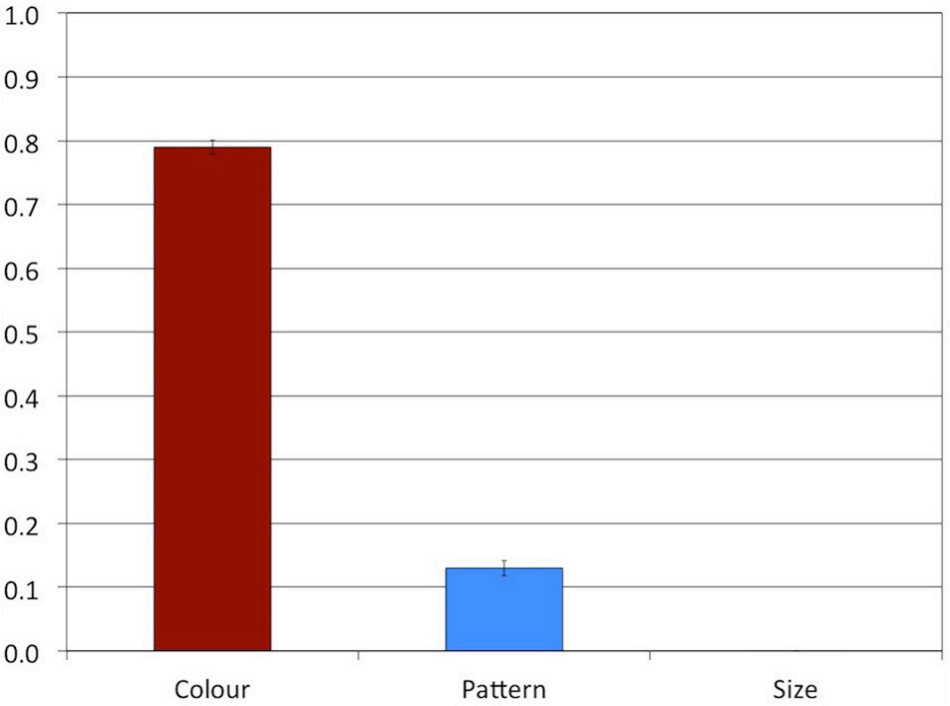
**Experiment 2: Proportions of overspecified referring expressions**. The error bars represent standard errors.

A comparison of Figures 4, 6 suggests that the patterns of results found in Experiments 1 and 2 were different, indicating that varying the three attributes within participants affected the proportions of overspecification in Experiment 1. A Kruskall-Wallis test indicated a main effect of Attribute in Experiment 2, *H*(2) = 35.98, *p* < 0.001. Stepwise stepdown comparisons revealed that the proportion of overspecification was significantly higher in the Color condition (*M* = 0.79, *SD* = 0.41, *MR* = 42.94) than in the Pattern condition (*M* = 0.13, *SD* = 0.34, *MR* = 22.06), *p* < 0.001. Although overspecification in the Size condition was at floor, it was still significantly lower (*MR* = 17.50) than in the Pattern condition, *p* = 0.037.

A Mann-Whitney test showed that the rate of pattern overspecification was significantly lower in Experiment 2 (*MR* = 14.33) than in Experiment 1 (*MR* = 22.67), *U* = 87.00, *z* = 2.61, *p* = 0.017, which indicates that the rate of pattern overspecification in Experiment 1 was affected by the tendencies to include or not include the other attributes. The rate of color overspecification was numerically higher in Experiment 2 (*MR* = 21.72) than in Experiment 1 (*MR* = 15.28), but this difference was only marginally significant, *U* = 220.00, *z* = 1.91, *p* = 0.07.

**Figure 7:**
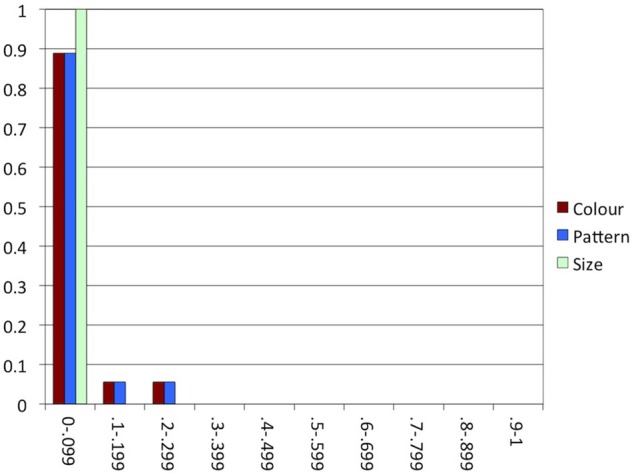
**Experiment 2: The proportion of participants (y-axis) in each range of proportions of alternations in each condition (x-axis)**.

As in Experiment 1, most participants alternated between producing and not producing overspecification within conditions in less than 10% of the trials, as indicated in Figure 7. That is, consistency was high again, which is in line with our expectation.

### 4.3. Discussion

The patterns of results found in Experiments 1 and 2 were clearly different, indicating that the rates of overspecification in Experiment 1 affected one another. In contrast to what was found in Experiment 1, where the rates of color and pattern overspecification were statistically indistinguishable, there was a large and highly significant difference between the Pattern and the Color conditions in Experiment 2. Although the rate of overspecification was significantly higher in the Pattern than in the Size condition in both experiments, the rate of pattern overspecification was closer to the rate of color than to the rate of size overspecification in Experiment 1, while it was the other way around in Experiment 2. A significant difference between the two Pattern conditions in Experiments 1 and 2 suggests that the production of color overspecification in Experiment 1 triggered the production of pattern overspecification. We found no evidence, on the other hand, that color overspecification *decreased* due to a tendency to *not* produce size overspecification: although the rate of color overspecification was numerically higher in Experiment 2 than in Experiment 1, this difference did not reach significance.

Experiment 2 indicates that the tendency to include color is stronger than the tendency to include pattern. Since both attributes are absolute, a possible explanation is that pattern is less salient than color. On the other hand, while the tendency to produce color overspecification may have triggered some participants to produce pattern overspecification, it did not trigger them to produce size overspecification. This may be because size is still less salient than pattern, but it may also be due to the fact that size is a relative attribute while both color and pattern are absolute.

In Experiment 3, we vary the three attributes within participants again, and we increase the contrast between big and small items, making size more salient. This enables us to investigate the respective effects of salience and absoluteness on the tendency to include attributes. In line with van Gompel et al. (2014), we might expect the rate of size overspecification to increase, indicating that salience is a factor in the tendency to include attributes and to produce overspecification. Furthermore, we expect an effect of absoluteness, resulting in a difference between color and pattern on the one hand, and size on the other hand, as in Experiment 1.

## 5. Experiment 3

In Experiment 3, we assess how salience and absoluteness contribute to the tendency to select attributes in referring expressions. As in Experiment 1, we vary color, pattern, and size within participants, but now increasing the salience of size, in order to find out whether this results in an increase in size overspecification compared to Experiment 1, which would indicate an effect of salience on overspecification. We also expect that there will remain a difference between the two absolute attributes (color and pattern) and size. Finally, we expect the degree of consistency within speakers again to be high.

### 5.1. Method

#### 5.1.1. Participants

We tested 18 participants (13 females, 5 males, mean age 21 years, range 18–29 years) similar to those in the previous experiments. None had participated in either of the previous experiments.

#### 5.1.2. Materials and design

**Figure 8:**
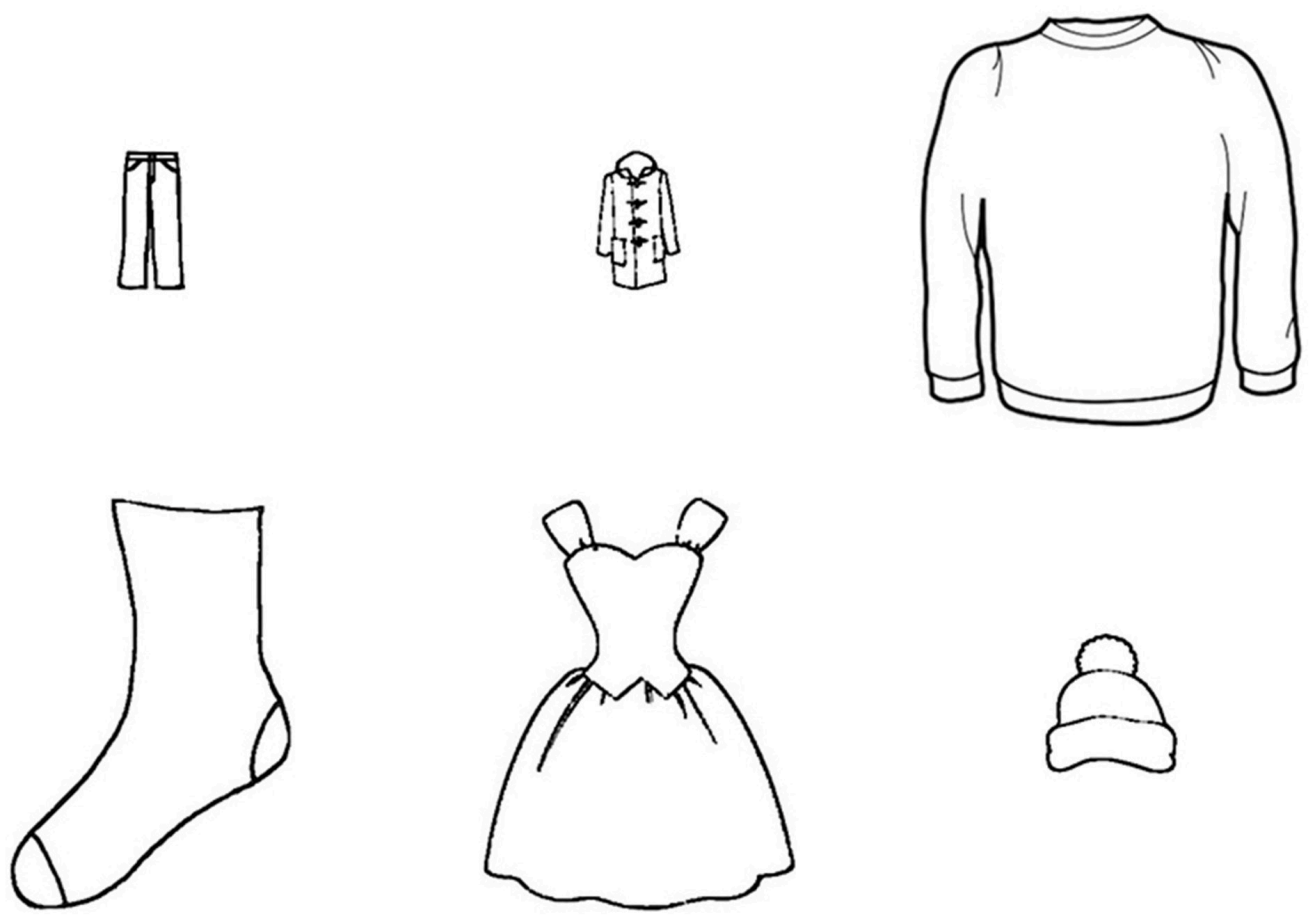
**An array in the Size condition in Experiment 3**.

In the Size condition, the ratio between big and small pictures was 3:1 instead of 3:2. An example of an array in the Size condition is shown in Figure 8. Otherwise, materials, design, and procedure were as in Experiment 1.

### 5.2. Results

All participants performed 36 critical trials each. Once, no response was given. The critical trials thus elicited 647 responses. Seven responses (1.1%) were removed from the analysis as in Experiment 1. The remaining 640 responses were annotated as in the previous experiments.

We conducted Experiment 3 to assess how salience and absoluteness contribute to the tendency to select attributes. Our first hypothesis was that an increase in salience of size would result in an increase in the rate of size overspecification from Experiment 1 to 3, indicating that salience contributes to this tendency. We also expected absoluteness to contribute, our second hypothesis being that there would still be a difference between color and pattern on the one hand, and size on the other hand (like in Experiments 1 and 2).

**Figure 9:**
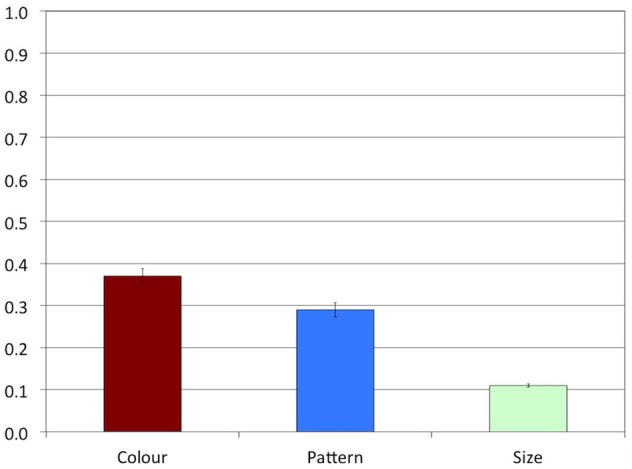
**Experiment 3: Proportions of overspecified referring expressions**. The error bars represent standard errors.

The proportions of overspecified referring expressions in each condition in Experiment 3 are shown in Figure 9. A Mann-Whitney test indicated that although the proportion of size overspecification was numerically higher in Experiment 3 (*M* = 0.11, *SD* = 0.31, *MR* = 20.17) than in Experiment 1 (*M* = 0.01, *SD* = 0.10, *MR* = 16.83), this difference was not significant, *U* = 129.00, *z* = 1.38, *p* > 0.1. Thus, our first hypothesis was not confirmed by the data.

In line with our second hypothesis, Figure 9 suggests that the patterns of Experiments 1 and 3 were globally similar, with overspecification being produced more often in the Color and the Pattern conditions than in the Size condition. Two additional Mann-Whitney tests confirmed that there was no significant difference between Experiments 1 and 3 for Color (*MR* = 20.72 vs. *MR* = 16.28, *U* = 122.00, *z* = −1.28, *p* > 0.1), and for Pattern (*MR* = 20.08 vs. *MR* = 16.92, *U* = 133.50, *z* = −0.94, *p* > 0.1).

A Friedman's ANOVA indicated that there was a significant main effect of Attribute, χ^2^(2) = 19.58, *p* < 0.001. Stepwise stepdown comparisons showed that the difference between the Color (*M* = 0.37, *SD* = 0.48, *MR* = 2.56) and Pattern (*M* = 0.29, *SD* = 0.45, *MR* = 2.03) conditions was not significant, *p* > 0.10, as in Experiment 1, and that the difference between Pattern and Size (*MR* = 1.42) was marginally significant, *p* = 0.059.

Earlier, we found a significant difference between the two Pattern conditions in Experiments 1 and 2, while the difference between the two Color conditions was only marginally significant (see Section 4.2). We thus found evidence that in Experiment 1, the rate of pattern overspecification was affected by tendencies to include or not include other attributes, but no evidence for analogous effects on the rate of color overspecification. However, a Mann-Whitney test indicates that the proportion of color overspecification was significantly lower in Experiment 3 (*M* = 0.37, *MR* = 13.86) than in Experiment 2 (*M* = 0.79, *MR* = 23.14), *U* = 78.50, *z* = −2.71, *p* = 0.007, indicating that the rate of color overspecification, too, is affected by the way other attributes are treated.

**Figure 10:**
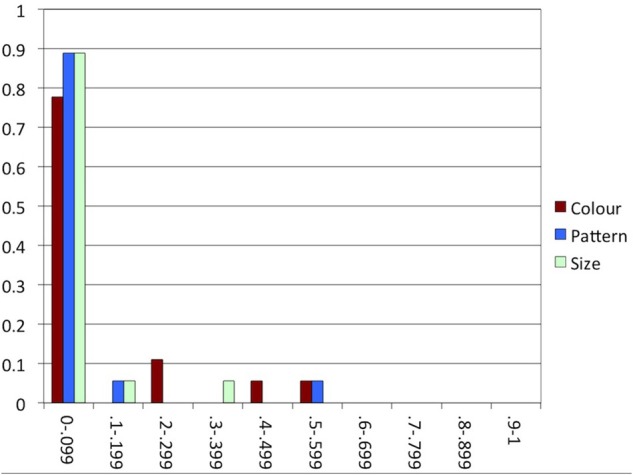
**Experiment 3: The proportion of participants (y-axis) in each range of proportions of alternations in each condition (x-axis)**.

As in the previous studies, most participants alternated between producing and avoiding overspecification within conditions in less than 10% of the trials, as indicated in Figure 10. That is, consistency was high again, which is in line with our expectation.

### 5.3. Discussion

Experiment 3 was conducted to assess how salience and absoluteness contribute to the tendency to produce overspecification of color, pattern, and size. We hypothesized that due to an increase in salience, the rate of size overspecification might increase, but that due to a difference in absoluteness, the rates of color and pattern overspecification would remain higher than the rate of size overspecification.

Our first expectation was not confirmed: there was no significant difference between the rates of size overspecification in Experiments 1 and 3. At first sight, this result does not seem to be in line with the findings of van Gompel et al. (2014), who did find a positive effect of increasing salience of size on size overspecification. However, as discussed in Section 2.2.1, there is a crucial difference between their experiments and ours: in their study, all items were of the same type but different sizes and colors, requiring either size or color for disambiguation between the target and the other objects, while in our study, all items were of different types and therefore it was never necessary to add a modifier to the noun. Thus, including size resulted in overspecification in our study, while in theirs, only including both color and size did. Even if both color and size were included in their study, however, size might still not be experienced as irrelevant by an addressee, because it did distinguish between objects of the same type. An eyetracking study conducted by Sedivy et al. (1999), which was touched upon briefly in Section 2.1, indicated that addressees expect speakers to use size adjectives only if the referent has a bigger or smaller counterpart in the context, whereas they do not have analogous expectations about the use of color adjectives. In this study, participants were shown arrays with, for example, a big and a small glass, a big pitcher, and a small key. Eye gaze patterns suggested that upon hearing “big,” participants inferred that the referent was the big glass rather than the big pitcher, whereas in a situation with a pink and a yellow comb, a yellow bowl, and a knife, they did not infer from hearing “yellow” that the referent was the yellow comb rather than the yellow bowl. These findings suggest that size adjectives are expected only if there is a relevant size contrast in the context, that is, if the referent is bigger or smaller than another object of the same type. There was such a relevant size contrast in the experiment conducted by van Gompel et al., where all objects in the array were of the same type, but not in our experiments, where all objects were of different types. Size overspecification would therefore violate an addressee's expectation, and possibly even lead to confusion, when produced in the visual contexts we used in our experiment, but not in the contexts used in van Gompel et al.'s study. This may urge speakers to avoid size overspecification when there is no relevant size contrast in the context, probably due to the fact that size is a relative attribute.

Alternatively, it is possible that the difference between van Gompel et al.'s findings and ours is due to the fact that the size contrast in their study was 5:1 whereas it was 3:1 in our study. As Figure 8 shows, however, the size contrast in our study was quite striking, which led one of the participants in a pilot study to ask for “the *very* small dress” (“de *hele* kleine jurk”) the first time when she came across a trial in which a small object was the target. We therefore think it unlikely that participants in our study did not include size because it was not sufficiently salient.

The absence of a significant effect of salience on size overspecification and the difference between our results and those found by van Gompel et al. suggest that absoluteness is an important factor in attribute selection: even if size is made salient, size overspecification is produced infrequently. This suggestion is in line with our expectation that due to the difference in the absoluteness dimension, the rate of size overspecification would remain lower than the rates of color and pattern overspecification. Although the difference between pattern and size was only marginally significant, we did find that the pattern of results in Experiment 3 was globally similar to the one in Experiment 1, where this difference was highly significant. None of the three conditions in Experiment 3 was significantly different from the corresponding conditions in Experiment 1. Besides, in both experiments, proportions of overspecification in the Color and Pattern conditions were statistically indistinguishable, and they were numerically closer to each other than either of them was to the Size condition. All in all, this suggests that absoluteness indeed contributes to the tendency to include certain attributes but not others.

If the low frequency of size overspecification in Experiment 3 is indeed due to the fact that the contrast on this relative attribute was irrelevant, this may also explain why the rate of color overspecification in Experiment 3 was so much lower than in Experiment 2. We know from the previous experiments that speakers strongly tend to behave consistently, treating similar attributes in a similar way. In Experiment 1, this resulted in the majority of participants including both color and pattern but not size, which was different from the other two in being relative and low in salience. The high salience of size in Experiment 3, however, may have led participants to treat all three attributes similarly, since all of them were salient, either including them all or including none of them. Since including them all would lead to the unnecessary and irrelevant mention of a relative attribute, the majority of the participants may have been triggered to produce no overspecification at all.

It might be noted that, as in the previous experiments, our manipulation of the size of the pictures was independent of the proportions among the objects that the pictures represent: for example, a dress is normally much larger than a sock. Because people are so experienced in interpreting pictures and their sizes, which are not always proportional to real life sizes, we assume that our participants will have had no problem interpreting the size of the pictures in the arrays. Letting go of real life proportions was inevitable in the light of our purpose, namely, to compare the rates of overspecification of size with the other two attributes. In many other studies (such as van Gompel et al.'s), size differences are indicated by representing several objects of the same type in different sizes (for instance, a small candle and several larger candles). As discussed in Section 2.2.1, this is suitable when the *competition* between size and other attributes is investigated: how likely are speakers to include size when including either size or color is sufficient? In that situation, overspecification only arises when both size and color are included. In the present study, however, we are interested in a comparison between overspecification of different attributes, including size. To investigate this, it is necessary that the target object is unique in a display and that it differs in size from different objects. As it is hard, if not impossible, to indicate in a realistic way that a sock is small *for a sock* by exploiting the proportion between the sock and a dress, especially if the ratio between big and small pictures is fixed, we decided to abstract from the natural sizes of the objects represented. The fact that size overspecification was often produced in Experiment 4 (see Section 6.2), in which the same displays were used, indicates that it is unlikely that participants were confused by the “unnatural” size differences between the pictures.

Experiment 3 shows that size overspecification is produced infrequently if there is no relevant size contrast in the visual context, even if size is made highly salient. In Experiment 4, we investigate whether there are nevertheless circumstances that *do* trigger size overspecification, even if there is no relevant size contrast. As speakers show a tendency toward consistency, triggering the mention of the three attributes is likely to result in an increase in the rates of overspecification of all attributes, including size.

## 6. Experiment 4

In Experiment 4, we investigate circumstances that may trigger size overspecification, by introducing non-critical trials which require speakers to include color, pattern, or size in order to yield a unique description. Since participants in previous studies were found to show a strong tendency toward consistency, we expect the non-critical trials to trigger mentioning the three attributes, yielding an increase in color and pattern in comparison with Experiment 3, and also, for the first time, the occurrence of size overspecification, even though there is no relevant size contrast present in the visual context.

### 6.1. Method

#### 6.1.1. Participants

We tested 20 participants (16 females, 4 males, mean age 22 years and 10 months, range 18–28 years) similar to those in Experiment 1^8^. None had participated in any of the previous experiments.

#### 6.1.2. Materials, design, and procedure

The critical pictures used in Experiment 3 were now used both as critical and non-critical pictures. The pictures that were used as fillers in the previous experiments were not used here. Otherwise, materials and procedure were as in the previous experiments.

As in Experiment 3, attribute was manipulated within participants. Non-critical trials were now included to trigger the use of modifiers. They were identical to critical trials, except that one of the garments shared the target's type (but not its value). For example, when the target was a big sock, then there was also a small sock in the array. In this context, omitting a size modifier (“Click on the sock”) would result in underspecification, which we know from a variety of studies to be rarely produced (e.g., Engelhardt et al., 2006; Arts et al., 2011b; Koolen et al., 2011; Davies and Katsos, 2013). Additionally, in half of the trials discriminatory power was increased to make the target value more salient and hence increasing the probability that speakers would include size modifiers even in the critical trials. In half of the trials, as in the previous experiments (LowDist), the target shared its value with two of its distractors (see Figures 1–3), whereas in the other half (HighDist), it did not share its value with any of them, increasing this value's salience. For example, if the target in the HighDist condition was blue, the five other pictures were green.

All 36 variants of each picture acted as the target of a critical trial twice: they acted as target once in the LowDist condition and once in the HighDist condition. They also acted as the target of a non-critical trial twice, yielding a total of 144 trials. Six additional trials were included for practice.

### 6.2. Results

All participants performed 72 critical trials each. The critical trials elicited 1440 responses, 45 of which (3.1%) were excluded from the analysis as in Experiment 1. The remaining 1395 were annotated as in Experiment 1.

**Figure 11:**
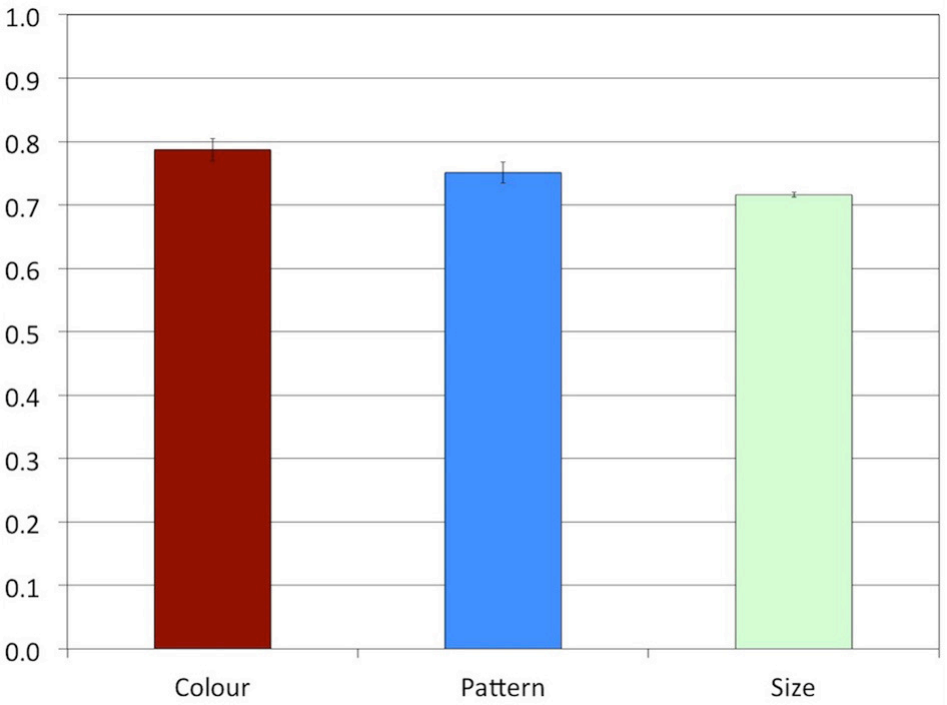
**Experiment 4: Proportions of overspecified referring expressions**. The error bars represent standard errors.

Experiment 4 was conducted to answer the question whether even size overspecification is triggered by mentioning color, pattern, and size. Additionally, in half of the critical trials (HighDist condition), we increased the salience of the target's value by making it unique in the array. The proportions of overspecified referring expressions in each condition are shown in Figure 11. In all conditions, including the Size condition, the proportion of overspecified referring expressions was now strikingly high, namely between 0.7 and 0.8. A comparison with the results of Experiment 3, presented in Figure 9, indicates an increase in the rate of color and pattern overspecification, and, crucially, also of size overspecification.

A Wilcoxon Signed Rank test was conducted first, to find out whether discriminatory power had an effect on overspecification. This turned out not to be the case, *z* = 1.28, *p* = 0.20, *r* = 0.29. Hence, the HighDist and the LowDist conditions were collapsed in all subsequent analyses.

Indeed, a Mann-Whitney test confirmed that the difference between Experiments 3 and 4 was highly significant for the Size conditions (*MR* = 11.11 vs. *MR* = 27.05, *U* = 331.00, *z* = 4.54, *p* < 0.001), and also for the Color (*MR* = 13.50 vs. *MR* = 24.90, *U* = 288.00, *z* = 3.26, *p* = 0.001) and the Pattern conditions (*MR* = 14.14 vs. *MR* = 24.32, *U* = 276.50, *z* = 2.97, *p* = 0.004).

Finally, a Friedman's ANOVA indicated that there was a significant main effect of Attribute in Experiment 4, χ^2^(2) = 11.81, *p* = 0.003. Pairwise comparisons indicated that the differences between Color (*MR* = 2.40) and Pattern (*MR* = 2.08) and between Pattern and Size (*MR* = 1.52) were not significant, *p* > 0.08 for both comparisons, while the difference between Color and Size was significant, *p* = 0.006.

**Figure 12:**
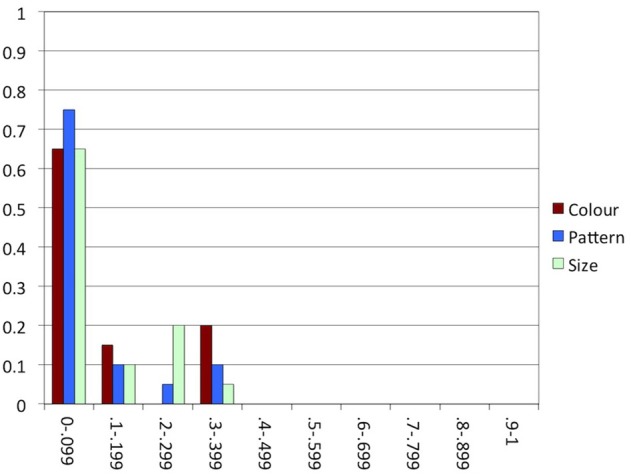
**Experiment 4: The proportion of participants (y-axis) in each range of proportions of alternations in each condition (x-axis)**.

As indicated in Figure 12, consistency was high, as in all previous experiments. In line with our expectation, the majority of the participants produced or avoided overspecification most of the time in all conditions.

### 6.3. Discussion

Experiment 4 shows that the strong tendency not to produce size overspecification that we found in our previous experiments can disappear almost entirely when mentioning color, pattern, and size is triggered. Although even in this experiment, more overspecification was produced in the Color than in the Size condition, the proportion of size overspecification strongly increased due to the non-critical trials, which required size modifiers, and it was very close to the proportions of overspecification in the Color and Pattern conditions, which were also significantly higher than the proportions of their counterpart conditions in Experiment 3.

To conclude, Experiment 4 provides evidence that overspecification, even of size, can be triggered under certain circumstances, due to a general tendency to behave consistently. Speakers thus do not necessarily avoid overspecification of a relative attribute, even if there is no relevant contrast on this attribute in the visual context.

## 7. General discussion

In this paper, we investigated the tendencies to produce color, pattern, and size overspecification. We compared rates of overspecification of the three attributes, focusing on the role of salience, absoluteness, and consistency. Since color and pattern are salient and absolute whereas size is relative and often less salient, we hypothesized that speakers would produce more color and pattern overspecification than size overspecification. Experiment 1, which had a within-participants design, confirmed this expectation: speakers produced substantial rates of color and pattern overspecification, which were very similar to each other, but almost no size overspecification.

Experiment 2 indicated, however, that in Experiment 1, pattern was treated similarly to color because the rates of overspecification affected one another: when varying the attributes between participants, the proportion of pattern overspecification was low, while the proportion of color overspecification was high. The tendency to select pattern is thus less strong than the tendency to select color. As both are absolute attributes, a possible explanation for this finding is that pattern is less salient than color. We concluded that in Experiment 1, the tendency to produce color overspecification probably stimulated the production of pattern overspecification, which is likely to be due to the fact that the two attributes are absolute and more salient than size. A comparison between Experiments 2 and 3, in which the three attributes were manipulated within participants again, indicated that the rates of overspecification of the three attributes can also affect one another in a different way: the rate of color overspecification was significantly lower in Experiment 3 than in Experiment 2. A plausible explanation is that the tendency *not* to include size triggered some participants to not include color either. In sum, Experiment 2 shows that the rates of overspecification of different attributes can affect one another due to a tendency toward consistency.

Experiment 3 was conducted to assess how salience and absoluteness contribute to the tendencies to select attributes. As in Experiment 1, attribute was manipulated within participants, but size was now made more salient by increasing size contrast. This manipulation did not result in a significant increase in size overspecification, however, and the patterns found in Experiments 1 and 3 were globally similar. In contrast to our findings, van Gompel et al. (2014) found that an increase in size contrast made speakers stop preferring color over size. Importantly, the size contrast in their study was relevant: when the referent was a small candle, there were also large candles in the array. In our study, in contrast, the referent was always unique, and the size contrast was therefore not relevant. Thus, an increase in salience can trigger selection of size, as van Gompel et al. show, but our study shows that salience is not *enough* to trigger size selection. The fact that a relevant contrast in the context seems to be crucial for including size suggests that size overspecification is infrequent because size is a relative attribute, indicating that absoluteness is a factor in attribute selection. This was supported by the fact that the pattern of results found in Experiment 3 was globally similar to the one in Experiment 1, where color and pattern were treated similarly, and differently from size, even though the difference between pattern and size was only marginally significant in Experiment 3.

In Experiment 4, finally, we found that even size overspecification can be triggered by mentioning color, pattern, and size, even though there was no relevant size contrast present in the critical trials. This finding is in line with Goudbeek and Krahmer (2012), who found that the selection of dispreferred attributes can be primed. It shows that the strong tendency toward consistency that was also found in the other three experiments can even lead to overspecification of attributes which otherwise do not tend to be included redundantly.

In many earlier studies investigating consistency in reference production, speakers appeared to have good reason to switch to a different construction: in Brennan and Clark (1996) and Van Der Wege (2009), the modified or otherwise highly specific terms that had been used before in the discourse would normally be dispreferred in the new context, and the attributes primed in Goudbeek and Krahmer (2012) are known to be normally dispreferred, too. The arrays used in critical trials in our experiments, in contrast, were highly similar, providing little reason for alternating between overspecification and minimal specification within conditions. This is especially clear in Experiment 2, where for each individual participant, objects in all arrays varied in the same attribute. Indeed, comparing Figures 5, 7, 10, 12 suggests that consistency was highest in Experiment 2. In the other experiments, where attribute was manipulated within participants, the alternation of the three attributes may have enhanced alternating between including and not including attributes within conditions.

As was stated in the Introduction, we are neutral as to what mechanisms underpin the tendency toward consistency in reference production in our experiments, and our study was not meant to settle the debate on those mechanisms. Still, it is worth pointing out that we think it most likely that the consistent behavior we found was due to priming. Although it is not impossible that our participants sought to establish conceptual pacts with their imaginary hearer, experimental studies suggest that effects of common ground considerations are so subtle that they can only be detected when the experimental set-up is sufficiently natural. For example, Brown and Dell (1978) seemed to show that interlocutors do not routinely take into account the common ground when telling stories, by conducting an experiment in which a naive participant interacted with a confederate. When replicating the experiment with pairs of two naive participants, however, Lockridge and Brennan (2002) were able to show that interlocutors did take into account the common ground after all. Since in our experiments no hearer was present at all, it is unlikely that the strong tendency toward consistency was due to the rather subtle effects of considerations of common ground. It is more plausibe that speakers primed themselves to include attributes previously included and reuse constructions. Whatever the underlying mechanisms are, the finding of such a strong tendency toward consistency has clear implications for the way experimental studies of referential behavior should ideally be designed. Our experiments show that decisions about the design, with respect to the conditions, and the non-critical trials have a significant effect on the results.

The present study has implications for the modeling of referring expression production, as is aimed at in the field of Referring Expression Generation (REG), which is a subfield of computational linguistics. REG models typically consist of an algorithm which generates a referring expression which distinguishes the referent from all other objects in a given context. The output of the algorithms are often evaluated against human-produced referring expressions. It was Pechmann's (1989) study, discussed in Section 2.2.2, which inspired Dale and Reiter (1995) to propose their now classic Incremental Algorithm, which selects attributes incrementally and in a predefined order (a “preference order”). Thus, the algorithm incorporates Pechmann's main finding, namely, that some attributes (such as color) are preferred and therefore selected before others (such as size). The Incremental Algorithm is very influential because it is conceptually and computationally simple, and hence efficient and easy to implement. However, there are several problems with this and related, more recent algorithms (Gatt et al., 2011; Krahmer and van Deemter, 2012).

First, the Incremental Algorithm is under-determined: it does not contain a procedure for finding a preference order (Krahmer and van Deemter, 2012). One way to overcome this problem is to collect production data which indicate what attribute preferences human speakers show when they produce referring expressions. Our study not only shows that color is preferred over pattern and that pattern is preferred over size, but also how salience and absoluteness contribute to those preferences. A second and more important problem is that the Incremental Algorithm is deterministic: in a given situation, it will always produce the same referring expression (Gatt et al., 2011). This is at odds with our finding that there is considerable variation across speakers (see also e.g., Viethen and Dale, 2010). Moreover, the Incremental Algorithm does not take into account the referring expressions that have been produced before in the discourse context. As was discussed in Section 2.3, however, more recent learning models that are able to align with their own previously produced referring expressions have been found to outperform models that do not take into account previously produced referring expressions (Viethen et al., 2014). Importantly, our findings indicate that including one attribute (such as color) can lead speakers to include another attribute (such as pattern), and that *not* including one attribute (such as size) can lead to not including another attribute (such as color and pattern). Modeling this behavior requires a selection procedure that is much more fine-grained than the procedure of the Incremental Algorithm and related algorithms.

Our study indicates that attributes vary in how likely they are to be selected when modification is not necessary. Speakers tend to include color, which is highly salient as well as absolute. The tendency to include pattern is less strong. Since pattern is like color in being absolute, this may suggest that pattern is less salient than color, and that salience is an important factor in the tendency to produce color overspecification, as proposed by Arts et al. (2011a), Gatt et al. (2013), and Koolen et al. (2013). Finally, our study shows that overspecification of size is rare when there is no relevant size contrast in the context, even if size is highly salient. The fact that the presence of a relevant size contrast matters strongly suggests that absoluteness is an important factor in the production of color overspecification, which has been argued before by Pechmann (1989) and Belke and Meyer (2002). However, even size overspecification can be triggered by mentioning the three attributes. In sum, our study indicates that color overspecification is more likely to occur than pattern overspecification because color is more salient than pattern, and much more likely than size overspecification because color is absolute while size is relative.

## 8. Ethics approval

This study was carried out in accordance with the recommendations of the Protocol Ethische Toetsing van Onderzoek (Protocol Ethical Approval of Research), Ethische Toetsingscommissie Geesteswetenschappen (Ethical Committee Faculty of Arts). All subjects gave written informed consent in accordance with the Declaration of Helsinki.

### Conflict of interest statement

The authors declare that the research was conducted in the absence of any commercial or financial relationships that could be construed as a potential conflict of interest.

## Footnotes

^1^Size is usually considered to be a relative attribute because in experimental studies of reference, speakers refer to size by using gradable adjectives like “big” and “small,” and not absolute measures such as centimeters.

^2^This is not strictly speaking true, as color perception is in fact highly sensitive to various features of the visual context. However, colors used for experimental stimuli are almost always bright, saturated colors that are highly typical for the color categories they fall into, being minimally sensitive to the context, rendering color practically an absolute attribute.

^3^To be precise, the discriminatory power of a referent's attribute is computed by dividing the number of competitors (the objects in the visual context other than the referent) that do not share the attribute with the referent by the total number of competitors.

^4^Stimulus images courtesy of Michael J. Tarr, Center for the Neural Basis of Cognition and Department of Psychology, Carnegie Mellon University, http://www.tarrlab.org/. In some cases, colors were adjusted or images were mirrored.

^5^This means we did not take into account *all* occurrences of overspecification. Color was sometimes included in the Pattern condition (*n* = 9) or in the Size condition (*n* = 2), but we did not count these cases as color overspecification. Doing so would not have yielded a fair comparison between the attributes because only pictures in the Pattern condition had patterns, while all pictures had a color. Moreover, patterns in line drawings are only there by the grace of color contrast.

^6^Data from eight additional participants were collected but not analyzed because they were instructed incorrectly (*n* = 4), because they received the wrong practice trials (*n* = 1), or because they failed to produce definite descriptions (*n* = 3).

^7^Participants never mentioned color in the Pattern or Size conditions, as happened sometimes in Experiment 1.

^8^Data from two additional participants were collected but not analyzed because they did not follow the instructions (*n* = 1) or because their age exceeded the upper age bound of 35 (*n* = 1).
